# An unusual cause for a painful Birmingham hip resurfacing

**DOI:** 10.1308/003588413X13511609955454

**Published:** 2013-01

**Authors:** KA Islam, SM Blake

**Affiliations:** South Devon Healthcare NHS Foundation TrustUK

**Keywords:** Hip arthroplasty, Postoperative pain, Peripheral vascular disease

## Abstract

The painful total hip arthroplasty requires careful evaluation and investigation. This is usually focused on the prosthesis and adjacent anatomical structures. We present a case report of a 64-year-old man who had a Birmingham hip resurfacing procedure for primary osteoarthritis. His hip pain worsened following the procedure and was under systematic investigation for this. Subsequent investigation for vascular disease revealed a total infrarenal aortic occlusion. An aortobifemoral bypass improved the hip pain and function dramatically, and the patient now has an excellent quality of life.

Total hip arthroplasty is a widespread and successful treatment for arthritic hip pain. Although postoperative function and satisfaction are high, there will be instances of pain following surgery. Targeted investigation will help to elucidate the diagnosis – by inclusion or exclusion. The clinician may logically investigate the implant or problems associated with surgery. However, when these common diagnoses are systematically excluded, more obscure causes of pain must be considered.

## Case history

A 64-year-old man presented with hip pain to our orthopaedic outpatient clinic. The pain was a dull ache at most times but much worse on walking for more than around 50 metres. It was centred on the right hip and radiated to the lateral thigh and buttock. He also suffered from left hip pain of a similar nature, which was not as severe. He felt that his hip pain was worsening and now affecting his quality of life. He was independently mobile without walking aids. He had been on prednisolone and then methotrexate for a number of years previously for inflammatory arthritis. He had a previous history of ischaemic heart disease.

**Figure 1:**
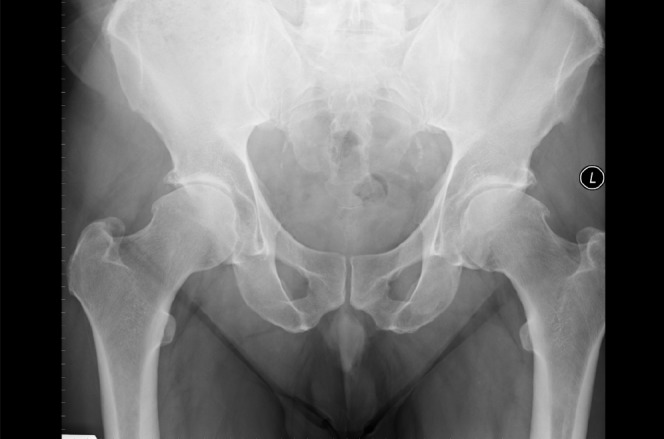
Anteroposterior x-ray of the pelvis with bilateral hip osteoarthritis

**Figure 2:**
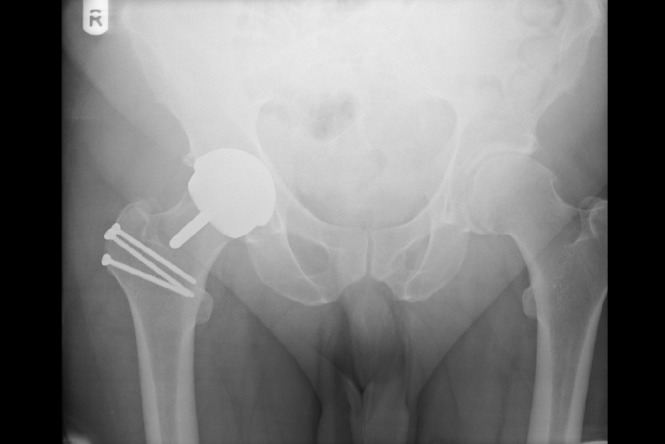
Post-operative x-ray following hip resurfacing

Plain x-ray images of the hip were consistent with osteoarthritis (Fig 1). A Birmingham hip resurfacing was performed via a lateral trochanteric osteotomy approach (Fig 2).

Postoperative mobilisation was poor, limited by pain in the hip and thigh on exercise. At the six-week review, the patient’s mobility had only improved slightly. Although his wounds had healed well, he could only mobilise with crutches. He was limited by aching pain in the operated hip. The quadriceps femoris was noted to be wasted and a provisional diagnosis of femoral nerve neurapraxia was made. He was encouraged to fully weight bear and continue mobilising. At eight months, his osteotomy screws were removed owing to continued trochanteric-based pain. However, his symptoms failed to improve.

**Figure 3:**
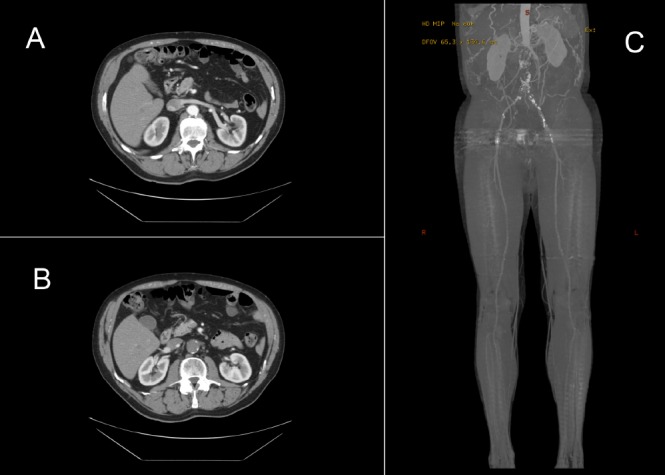
Computed tomography angiography slices with contrast showing patent aorta above renal vessels (A), complete occlusion of aorta (B) and reconstruction with complete occlusion of infrarenal aorta (C)

During further workup, the patient was noted to have reduced peripheral vascular supply. The ankle brachial pressure index was measured at 0.42 on the right and 0.44 on the left. Duplex ultrasonography of his aorta and lower limb vasculature showed a complete occlusion in the aorta with no flow in either common iliac artery. There was retrograde flow in the internal iliac artery on the left and external iliac artery on the right. There was no significant femoropopliteal disease, with three-vessel run-off in the calves. Computed tomography angiography was performed, which confirmed complete aortic occlusion at the infrarenal region extending to the common iliac artery on both sides (Fig 3).

The patient was advised by vascular surgeons to have an aortobifemoral bypass, which was performed. His hip pain and exercise tolerance were markedly improved. He now walks without a limp or walking aids.

## Discussion

Evaluation of hip pain after total hip arthroplasty can be challenging^1^ and requires systematic investigation.^2^ Thorough history and examination will guide the clinician through a myriad of differential diagnoses, broadly divided between intrinsic and extrinsic causes of pain. Intrinsic causes include infection, osteolysis, mechanical loosening, tip of stem pain (modulus mismatch), stress/periprosthetic fracture and inflammatory causes (eg bursitis or tendonitis). Extrinsic causes include nerve injury or irritation, causalgia or complex regional pain syndrome, referred pain (eg from lumbar spine pathology), peripheral vascular disease, metabolic conditions (eg Paget’s disease or osteomalacia) and malignancy or metastases.

In the case discussed, the initial diagnoses considered were femoral nerve neurapraxia, pain from the trochanteric osteotomy fixation screws and trochanteric bursitis. Ultimately, the cause of the pain was found to be vascular in origin and resolved following bypass surgery.

Lower limb ischaemia typically presents with intermittent claudication (ie cramping or burning pain brought on by exercise and relieved by rest). Unusual presentations of lower limb ischaemia have been reported (eg paraesthesia only in the lower limb on exercise^3^ or even stress incontinence on exercise in a patient with aortic occlusion).^4^ Ischaemic pain may be masked by soft tissue complaints, thereby presenting with a confusing picture.^5^ Claudication pain arising in the buttock region is a recognised symptom of internal iliac artery stenosis.^6^ In an already partly ischaemic limb, it has been suggested that following total hip replacement, further damage to collaterals may occur, leading ultimately to worsening ischaemic pain.^7^

## Conclusions

Our case highlights the difficulty of assessing the painful hip arthroplasty. It is reasonable to investigate causes of pain arising from the implant or complications of surgery in the first instance. However, when common causes of hip pain have been excluded, the case must be re-evaluated to consider less obvious causes of pain.

In our patient, an ischaemic cause of pain following a Birmingham hip resurfacing was found. This was successfully treated with vascular bypass surgery with a complete resolution of the symptoms.
